# Monitoring soil for sustainable development and land degradation neutrality

**DOI:** 10.1007/s10661-017-6415-3

**Published:** 2018-01-04

**Authors:** Gergely Tóth, Tamás Hermann, Manuela Ravina da Silva, Luca Montanarella

**Affiliations:** 1 Georgikon Faculty, Department of Crop Production and Soil Science, University of Pannonia, Keszthely, Hungary; 2 Centre for Agricultural Research, Institute for Soil Science and Agricultural Chemistry, Hungarian Academy of Sciences, Budapest, Hungary; 3 Global Environment Facility, Washington, DC USA; 4 European Commission, Joint Research Centre, Directorate D - Sustainable Resources, Ispra, Italy

**Keywords:** Land degradation, Monitoring, SDGs, Nutrient cycling, Productivity

## Abstract

The adoption of the 17 sustainable development goals (SDGs) listed in the 2030 Agenda for Sustainable Development by the United Nations urged the scientific community to generate information for planning and monitoring socioeconomic development and the underlying environmental compartments. SDGs 2, 3, 6, 11, 13, 14, and 15 have targets which commend direct consideration of soil resources. There are five groups of SDGs and assigned SDG indicators where soil plays a central role. Frameworks of soil-related sustainable development goals and related indicators which can be monitored in current monitoring schemes are proposed.

The United Nations’ adoption of the 17 sustainable development goals (SDGs), under the 2030 Agenda for Sustainable Development, urged the scientific community to generate sound information with the aim of supporting planning and monitoring of socioeconomic development interlinking with environmental sustainability dimensions (UN 2015). SDGs 2, 3, 6, 11, 13, 14, and 15 refer to targets which commend direct consideration of soil resources. For instance, food security (SDGs 2 and 6), food safety (SDG 3), land-based nutrient pollution of the seas (SDG 14), urban development (SDG 11), and sustainability of terrestrial ecosystem services (SDG 15) are all depending on the provision of ecosystem services where soil properties and functions play a key role to deliver these. In particular, SDG target 15.3 on land degradation neutrality mentions, *by 2030 to combat desertification, restore degraded land and soil, including land affected by desertification, drought and floods, and strive to achieve a land degradation-neutral world*. In addition, soils play an important role in mitigating and adapting to climate change (SDG 13). Further, SDGs 7 and 12 will indirectly rely on the availability of healthy soil resources. Regarding the remaining SDGs, linkages can be found to the sustainable management of soils to some extent (Keesstra et al. 2016).

The recent 48th session of the UN Statistical Commission revised the list of the global SDG indicators (UN 2017). The targets and indicators shown on Table 1 list soil properties and its functions. Currently, a soil-based indicator assigned to any of the soil-related SDGs is non-existent. The option of including such indicator is explicitly provided in the cases where disaggregation of indicators is relevant (UN 2017). In order to achieve the SDG goals based on soil resources, the relevance of including soil indicators from signaling to implementation stage is evident (Bouma and Montanarella 2016).

**Table 1:** Soil-related sustainable development goals and indicators

Soil-related sustainable development goal target	Soil-based/soil-related SDG indicator	Relevant soil function/property	Suggested minimum soil indicator to monitor
2.3 By 2030, double the agricultural productivity and incomes of small-scale food producers, in particular women, indigenous peoples, family farmers, pastoralists, and fishers, including through secure and equal access to land, other productive resources and inputs, knowledge, financial services, markets, and opportunities for value addition and non-farm employment.	2.3.1 Volume of production per labor unit by classes of farming/pastoral/forestry enterprise size	Biomass productivity	Nutrient cycling: OC, P, C/N ratio; soil hydraulic properties: OC, EC, bulk density, soluble Na; soil morphology; pH
2.4 By 2030, ensure sustainable food production systems and implement resilient agricultural practices that increase productivity and production, that help maintain ecosystems, that strengthen capacity for adaptation to climate change, extreme weather, drought, flooding, and other disasters, and that progressively improve land and soil quality.	2.4.1 Proportion of agricultural area under productive and sustainable agriculture	Biomass productivity	Nutrient cycling: OC, P, C/N ratio; soil hydraulic properties: OC, EC, bulk density, exch. Na; soil morphology, pH
3.9 By 2030, substantially reduce the number of deaths and illnesses from hazardous chemicals and air, water, and soil pollution, and contamination.	–	Concentration of hazardous elements	Concentration of hazardous elements, pH
6.4 By 2030, substantially increase water-use efficiency across all sectors and ensure sustainable withdrawals and supply of freshwater to address water scarcity and substantially reduce the number of people suffering from water scarcity.	–	Soil hydraulic properties	Bulk density, OC, EC, soil morphology
6.5 By 2030, implement integrated water resources management at all levels, including through transboundary cooperation as appropriate.	6.5.1 Degree of integrated water resources management implementation (0–100)	Soil hydraulic properties	Bulk density, OC, EC, soil morphology
11.3 By 2030, enhance inclusive and sustainable urbanization and capacity for participatory, integrated, and sustainable human settlement planning and management in all countries.	11.3.1 Ratio of land consumption rate to population growth rate	Biomass productivity, soil hydraulic properties	Nutrient cycling: OC, P, C/N ratio; soil hydraulic properties: OC, EC, bulk density, exchangeable Na; soil morphology
13.2 Integrate climate change measures into national policies, strategies, and planning.	–	Organic carbon content	OC
14.1 By 2025, prevent and significantly reduce marine pollution of all kinds, in particular from land-based activities, including marine debris and nutrient pollution.	–	Erodibility, N, P	Bulk density, OC, N, P, topsoil depth
15.3 By 2030, combat desertification, restore degraded land and soil, including land affected by desertification, drought, and floods, and strive to achieve a land degradation-neutral world.	15.3.1 Proportion of land that is degraded over total land area		Nutrient cycling: OC, P, C/N ratio; soil hydraulic properties: OC, EC, bulk density, exchangeable Na; soil morphology; topsoil depth, pH
15.5 Take urgent and significant action to reduce the degradation of natural habitats, halt the loss of biodiversity, and, by 2020, protect and prevent the extinction of threatened species.	–		Selected soil biodiversity indicators

The main question lies in what soil indicators should be considering and the monitoring methodologies behind. An analysis on the content of the SDGs and its indicators helps to clarify the level of integration of soils to these. There are five groups of SDGs and assigned indicators where soil plays a central role, namely:

1. explicitly include productivity (2.3, 2.4),
2. explicitly include soil degradation (15.3),
3. name soil in the SDG although no soil-based indicator has been proposed (3.9),
4. have direct relevance to soil resources with explicit reference to land resources but no reference to soil (11.3),
5. have direct relevance of soil to SDG without naming soil in SDG nor including soil-related SDG indicator (6.4, 6.5, 13.2, 14.1, 15.5).

Productivity is the soil property considered in several SDGs and incorporated into a broader concept of “agricultural productivity” term (SDGs 2.3, 2.4). Nevertheless, while agricultural productivity defines various factors and might also include components like animal husbandry and other segments, the fundamental factor of agricultural productivity is based on soil fertility (and climatic conditions). In addition, soil productivity loss is a central concept of land degradation (UNCCD 1994). Land degradation, in turn—apart from being a separate item among the SDGs (15.3)—refers also to the process leading to the reduction of soil water holding capacity and conductivity, loss of soil biodiversity, soil pollution, and/or nutrient load (SDGs 3.9, 6.4, 6.5, 14.1, 15.5). With regard to climate regulation (SDG 13.2), soil organic carbon (SOC) is considered the most relevant soil property.

To achieve the SDGs, there is an urgent need to assess and monitor the soil properties that impact soil productivity and the soil threats, with an emphasis on soil hydraulic properties, nutrient status, pollution, soil biodiversity, and SOC changes. An option is through the assessment of soil properties in the minimum datasets (Doran and Parkin 1996; Li et al. 2007). These include physical, chemical, and biological indicators of which standardized assessments and regular monitoring are carried out (Nortcliff 2002). However, current monitoring systems are not fully prepared to provide complete required information (Morvan et al. 2008; Tóth et al. 2017). Soil hydraulic properties (water retention and conductivity) and nutrient cycling are key indicators for soil productivity. In addition, vertical soil properties (horizons), underlying hydrology and topography, are also important. Soil texture on the other hand is the most basic soil physical attribute which determines hydraulic properties. However, as it remains unaltered over the medium term, monitoring focuses rather on other relevant attributes (i.e., OC, bulk density, salinity). These likewise will influence on the soil water status. OC plays a central role not only in soil water characteristics and climate control but also in the nutrient status of soil. Contamination and soil biodiversity remain separate for which the monitoring programs will need to include. Nevertheless, if these aspects of soil quality can be assessed and monitored, much of the SDGs can indeed be followed. Table 1 suggests the most relevant soil physical, chemical, and biological indicators that can potentially be applied within the framework of the SDG indicators.

Efforts are being made for improving soil monitoring systems, such as the European Union’s work gathering information on soil bulk density and soil biodiversity (Fernández-Ugalde et al. 2016) along with the potential of mass data gathering and big data on morphological properties and OC status. These are examples that will help enable the use of soil indicators to support the monitoring of the SDGs. In addition, there needs to be established a global indicator framework for monitoring progress in protecting soil resources and soil-based sustainable development.

The spatial extent of global and regional soil quality assets has to be considered and integrated into policies ranging from sustainable urbanization to agricultural development. Soil functions and degradation threats are manifold, and soil indicators are diverse. Monitoring soil and land changes for all these considerations is a challenge which needs to be addressed. The SDGs help to streamline the efforts of soil monitoring programs, which, combined with the new sources of soil information and extensive application of modern technological solutions can provide the needed data and information. Additionally, harmonization between different sampling and monitoring programs as well as between measurement standards and protocols (Nortcliff 2002; Morvan et al. 2008) still needs to be completed. Current efforts towards global harmonization of soil information, e.g., the upcoming global soil organic carbon map by the Global Soil Partnership (GSP FAO and ITPS 2017) remarks the fulfillment of these requirements being feasible. The next steps are to apply this valuable knowledge to fully align and support the achievement of the SDGs.
